# The Orthopedic Effects of Electronic Cigarettes: A Systematic Review and Pediatric Case Series

**DOI:** 10.3390/children9010062

**Published:** 2022-01-04

**Authors:** Maxwell Luke Armstrong, Nicholas Smith, Rhiannon Tracey, Heather Jackman

**Affiliations:** 1 Division of Orthopedic Surgery, Janeway Children’s Health and Rehabilitation Centre, Memorial University of Newfoundland, St. John’s, NL A1B 3V6, Canada; nicksmith@munmed.ca (N.S.); hrjackman@gmail.com (H.J.); 2 Division of Orthopedic Surgery, Schulich School of Medicine and Dentistry, Western University, London, ON N6A 5A5, Canada; rmt224@mun.ca

**Keywords:** e-cigarette, vaping, adolescent health, pediatric orthopedics

## Abstract

Electronic cigarette (EC) use is highly prevalent, especially in the adolescent population, where 29% of Canadian adolescents have used an EC in the past thirty days per national surveys. Our pediatric orthopedic referral centre observed a cluster of delayed unions of bone fractures in adolescents using ECs and present the case series here. We then asked whether electronic cigarettes impair bone healing or influence orthopedic outcomes. A PRISMA-compliant systematic review was carried out, which revealed no human clinical studies and a general paucity of evidence around ECs and musculoskeletal health. The existing experimental evidence relevant to orthopedics is summarized. The effect of ECs on the musculoskeletal system is poorly understood and is a target for further research.

## 1. Hypothesis-Generating Cases

### 1.1. Ankle Fracture While Roughhousing, Eighteen Weeks in a 15-Year-Old Male

A fifteen-year-old male with no known medical history sustained a closed, minimally displaced, simple oblique trans-syndesmotic lateral malleolus fracture with no widening of the mortise. There was a remote history of occasional cigarette smoking and the patient was a current user of electronic cigarettes (EC). The fracture was reduced and splinted in a peripheral Emergency Room and referred to our centre for definitive management.

**Figure 1:**
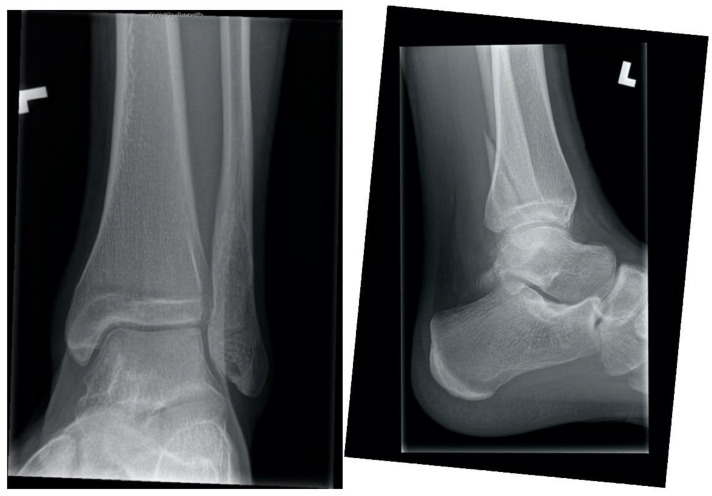
Twelve-week ankle radiographs demonstrating no radiographic union after fibula fracture in a pediatric electronic cigarette user.

Initial treatment was circumferential casting and non-weight bearing. At one-week post-injury X-rays confirmed near-anatomic alignment. At four weeks post-injury radiographs were equivocal for callus, the patient’s exam was reassuring, and a removable cast-boot was placed with progressive weight bearing. At eight weeks post-injury, there was pain at the fracture site and inability to bear full weight. The patient was counselled to discontinue EC use. Cast-boot immobilization with weight bearing as tolerated was continued. There was no clinical or radiographic change at twelve weeks post-injury (Figure 1). Delayed union was diagnosed in the absence of healing beyond the expected four to six weeks required to heal this injury [1]. At eighteen weeks post-injury the fracture was no longer symptomatic, and radiographs confirmed bony union.

### 1.2. Monteggia Fracture-Dislocation on a Motorcycle, Fifteen Weeks in a 14-Year-Old Male

A fourteen-year-old male presented with a closed, neurovascularly intact, displaced, and moderately comminuted diaphyseal ulnar fracture with associated posterolateral dislocation of the radial head, also known as a Monteggia fracture-dislocation. The injury occurred after ejection from an off-road motorcycle at moderate speed. There was an associated unstable C5/6 cervical spine fracture-dislocation and disc rupture with no neurological symptoms. There was no known medical history. The forearm fracture-dislocation was reduced and splinted in the trauma bay by pediatric orthopedics with near-anatomic alignment. Neurosurgery colleagues managed the spine injury with emergent anterior discectomy and fusion without complication from which he recovered well with no neurologic sequelae. There was no history of cigarette smoking, but the patient was a current user of e-cigarettes.

**Figure 2:**
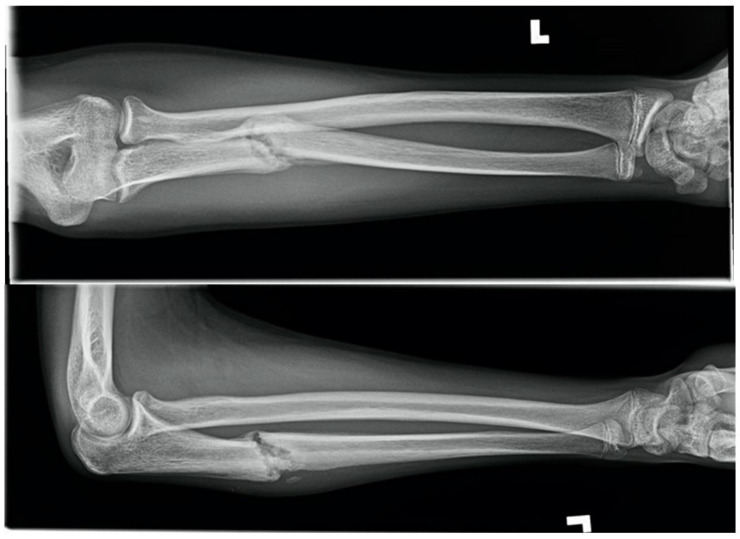
Eleven-week forearm radiographs demonstrating no union after Monteggia fracture-dislocation in a pediatric EC user.

At one-week post-injury radiographs demonstrated good alignment of the fracture and radio-capitellar joint. A long-arm circumferential cast was placed. At three weeks post-injury radiographs again demonstrated acceptable alignment and casting was continued. At seven weeks post-injury, there was no radiographic evidence of healing, and a removable cast was prescribed with instructions for twice daily non-weightbearing range of motion exercises. The patient was counselled to discontinue EC use. At eleven weeks post-injury there was continued pain at the fracture site with no radiographic evidence of healing (Figure 2). Delayed union was diagnosed as the expected healing time of a closed pediatric forearm fracture is approximately 5.5 weeks [1]. Fifteen weeks post-injury the patient had symptomatic improvement with resolution of fracture-site tenderness and radiographs showed evidence of bony union.

### 1.3. Forearm Fracture on a Trampoline, Eighteen Weeks in a 15-Year-Old Male

A fifteen-year-old male sustained closed, neurovascularly intact, displaced fractures of the radial and ulnar diaphyses with comminution of the radius. The mechanism of injury was a fall on an outstretched hand on a trampoline. The patient had no systemic comorbidity and no history of smoking but was a current user of electronic cigarettes. The fracture was splinted in a peripheral Emergency Room and was referred to our centre. The following day, the fractures were definitively treated without complication by open reduction internal fixation (ORIF) with plates and screws. The simple ulnar fracture was plated in compression mode, and the mildly comminuted radial fracture was approximated and plated in bridge mode.

**Figure 3:**
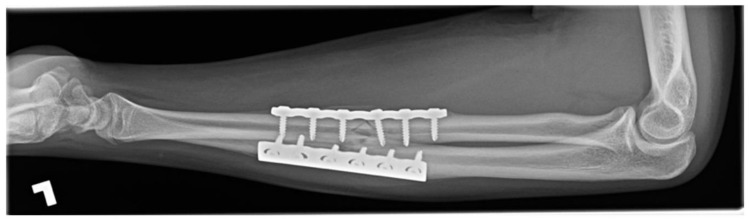
Twelve-week radiograph of an operatively treated both-bones forearm fracture in a pediatric electronic cigarette user. The ulna is healed, but the radius is not.

Five weeks after operative treatment splint immobilization was discontinued, and a functional brace was applied. Eight weeks after ORIF, radiographs demonstrated bony union of the ulna but no union of the radius fracture despite anatomic alignment. The patient was counselled to discontinue EC use. Again, at twelve weeks, there was no union of the radius fracture and the patient had ongoing symptoms (Figure 3). Delayed union was diagnosed. Eighteen weeks after ORIF the patient had clinically improved, radiographs demonstrated bony union, and treatment was discontinued.

## 2. Background and Rationale for Review

Impaired bone healing is rare in the pediatric population. A large Scottish study found 422 fracture non-unions in 161,100 fractures diagnosed in patients aged 0–18 over five years, for an overall incidence of 0.2–0.35% per fracture [2]. Risk factors for impaired bone healing in pediatric patients include specific fracture characteristics, local bone biology, systemic host health, increasing age, and orthopedic treatment tactics. Complications of impaired bone healing may include pain, deformity, reduced mobility, and unplanned surgery [1].

During a review of the fractures treated at our pediatric orthopedic referral centre, the above cases were noted as a cluster of difficult-to-heal fractures. Retrospectively, we observed the common exposure of e-cigarette use. During the ten months in 2018–2019 when these cases were managed, 330 fractures in patients aged 12 to 18 were treated at our centre. Three of these 330 fractures had delayed union, which are the three presented above. This led us to pose the question: Do electronic cigarettes affect bone healing?

These cases are presented without any inference of an association between ECs and bone healing and serve only as a hypothesis generator. The impaired bone healing in our three cases may be attributable to a variety of factors. For example, in Case 1, patient adherence to removable cast-boot immobilization of the fibula fracture is unknown. In Case 2, a higher energy mechanism, the biology of the ulnar diaphysis, and nonoperative treatment tactics may have contributed to impaired healing. An iatrogenic fracture gap created by a bridging plate and screw fixation of a comminuted radial shaft fracture in Case 3 could be a treatment-related non-union risk factor.

Electronic cigarettes (ECs) are pocket-sized devices that combine heat and the user’s inhalation to vaporize a solution to produce a cloud of vapor that mimics the experience of conventional cigarette smoking, often called ‘vaping’. The solutions are proprietary but are usually composed of water, propylene glycol, and glycerine, as well as additives of flavorings and nicotine in varying concentrations [3]. A proposed danger of ECs is the ability to achieve high nicotine doses by selecting a solution with high nicotine content and vaping more frequently [3]. The long-term health effects of these products are unknown, as are the actual chemicals delivered to the bloodstream in vivo after passing the solution over a superheated coil. The characterization of the ‘E-cigarette or vaping product use-associated lung injury’ (EVALI) has been a recent area of intense research focus and media scrutiny [4,5]. Significant attention has also duly been given to the diagnosis and treatment of facial blast injuries from exploding devices [6].

The popularity of ECs amongst Canadian adolescents is alarming [4]. The Canadian Student Tobacco, Alcohol, and Drugs Survey (CSTADS) 2018–2019 demonstrated that 29% of students in high school were current users and had vaped in the past 30 days, a rapid increase from prior years. Daily or occasional cigarette smoking had a 3% prevalence, which was stable [7].

Complicating the issue is the marketing of ECs, which usually suggests that they are a healthier, ‘cleaner’ alternative to cigarettes. This appeals to consumers who perceive a healthier option with a similar experience to smoking. ECs are marketed to adults as a smoking cessation option. A range of vapour flavours and modern device styling with USB charging have likely contributed to the concerning prevalence of use in the pediatric population [7].

It is tempting to equate the health effects of conventional cigarettes and ECs as they both deliver nicotine via inhalation and the user experience is similar. However, they are chemically dissimilar products except for nicotine. While the negative effect of tobacco smoking on bone healing is unequivocal, the contribution to this effect from the chemical nicotine specifically is still under investigation [8,9].

This study asked whether electronic cigarettes had a deleterious effect on bone healing and, more broadly, on orthopedic outcomes. We conducted a PRISMA-compliant systematic review and present the results below.

## 3. Systematic Review

### 3.1. Eligibility Criteria

All publications with potential relevance to the operative or non-operative practice of orthopedics in English or French were examined (no results were found in other languages). For completeness, human, animal, and experimental studies were considered from all levels of evidence.

### 3.2. Search Strategy and Sources

In March 2021, the PubMed, EMBASE, and Cochrane databases were searched for relevant publications. Each of these databases were interrogated with the basic input, ((e-cigarette) OR (vaping)) AND ((orthopedics) OR (surgery)). The extended search terms from PubMed are displayed in Table 1 as an example and no limits or filters were used. To search the grey literature, the OpenGrey database (www.opengrey.eu, accessed on 22 March 2021) was searched with the same terms. This strategy yielded 608 papers for consideration after removal of duplicates.

### 3.3. Study Selection and Data Colletion Process

Upon initial review of the results, it was obvious that our primary question of the effects of e-cigarettes on bone healing would not be answered by the current literature. There were no human studies of bone healing in EC users. We pursued our broader goal of collecting any literature pertaining to the effect of e-cigarettes on orthopedic outcomes. The corresponding author (M.L.A.) sorted and screened the initial 608 records for potential relevance to orthopedics based on the title and abstract. Records with titles indicating relevance exclusively to a non-orthopedic topic (i.e., smoking cessation, economics, sociology) were excluded. All potentially relevant abstracts were reviewed and excluded if there was no pertinence to the musculoskeletal system and ECs or EC vapour.

**Figure 4:**
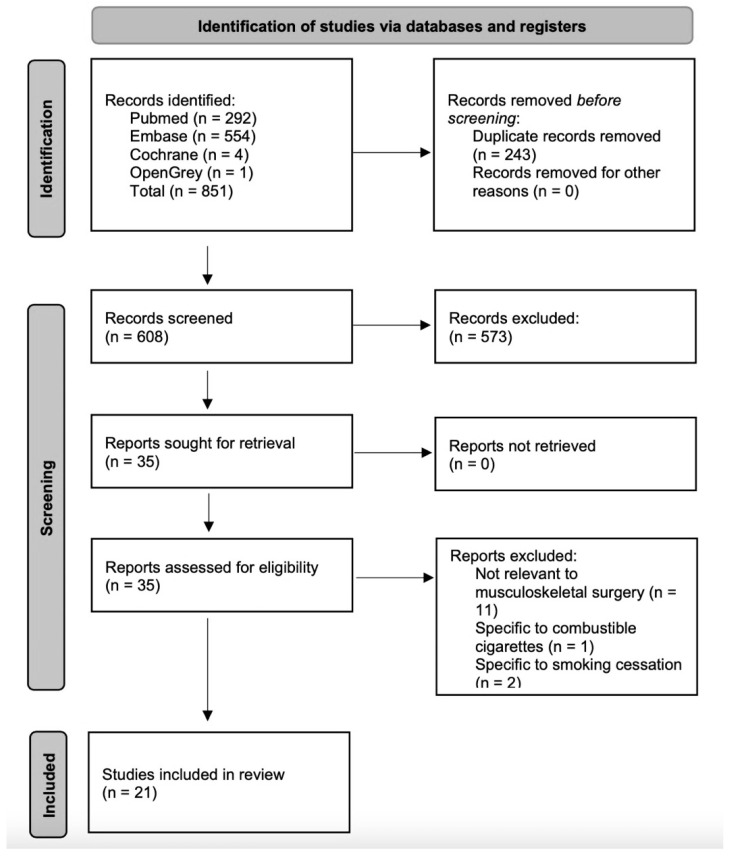
Systematic review flowchart.

Thirty-five potentially relevant records were then retrieved for manuscript assessment. No automation tools were used. The title, abstract, and manuscript were read and assessed for pertinence to orthopedics. Further exclusions were made on the basis of no relevance to orthopedics (*n* = 11), no relevance to electronic cigarettes (*n* = 1), and for a focus only on smoking cessation (*n* = 2). Publications were grouped for their topic/purpose and quality of evidence. The systematic review workflow is summarized in Figure 4.

This review protocol was submitted for registration on the International Prospective Register of Systematic Reviews (PROSPERO identification number: 299563).

## 4. Results

No Level 1 studies were found. No human clinical studies were found. Only six studies were found that were experimental, which are summarized in Table 2. In summarizing these studies, they clustered around three subtopics—basic science, musculoskeletal health, and wound healing. Data extraction was not applicable as no relevant human clinical studies on e-cigarettes were found.

Most publications were non-systematic reviews, briefs, and opinion letters. These are not discussed here but are cited in the references list per Table 2.

## 5. Discussion

Our systematic review highlights the paucity of evidence about the effect of ECs on orthopedic surgery and the musculoskeletal system in general. The papers that were found pertained to basic science, musculoskeletal health, and wound healing.

Pywell et al. published a human experiment on 15 subjects that demonstrated a reduction in hand microcirculation after inhaling a 24 mg nicotine e-cigarette but not after a 0 mg e-cigarette. They concluded the consumption of nicotine causes vasoconstriction [10]. Romagna et al. exposed fibroblast cell cultures to liquified solutions of EC vapor and combustible cigarette smoke and found the liquified smoke to be significantly more cytotoxic [11].

Kennedy et al. undertook an animal experiment with rats exposed to two cigarettes daily or the nicotine equivalent of electronic cigarette vapour for four weeks. The rats had Achilles tendon transection and repair performed and then the tensile strength of the repair was measured two weeks later. The EC cohort had a significantly lower load-to-failure versus the cigarette and control cohorts, which were not significantly different [12]. Reumann et al. exposed mice to EC vapour or the nicotine equivalent of cigarette smoke daily for six months and then quantified bone strength parameters. In this study, the cigarette smoke significantly decreased bone density and bending strength compared to EC vapour and the control group, which were not significantly different [13].

In 2019, Rau et al. raised and then repaired skin flaps on a rat model exposed to low-dose EC vapour, high-dose EC vapour, or combustible cigarettes for four weeks. Five weeks after surgery flap survival was significantly decreased in the exposure groups versus the control group, but not within the three exposure groups [14]. This rat experiment was essentially repeated by Troiano et al., who also found no significant difference in flap necrosis between EC vapour- or cigarette-exposed rats but did find a significant increase in necrosis in these exposed groups versus the control [15].

Other groups have published non-systematic reviews and expert briefs of the topic, including Amaro et al. in *JBJS Reviews* in 2019. They compared and contrasted ECs with combustible cigarettes and overviewed the orthopedic issues [17]. Fiani et al. similarly overviewed the topic with a focus on the spine and molecular toxicology [18]. Nicholson et al. recently published an expert brief on the musculoskeletal biochemistry of vaping in *Bone and Joint Research* [16].

This systematic review is the most current collection of knowledge about the orthopedic effects of electronic cigarettes. This review is severely limited in its ability to draw any clinically relevant conclusions about the orthopedic effects of electronic cigarettes based on the current body of evidence. A further limitation is that a single researcher reviewed the searched publications for suitability, which may introduce bias in judgement or clerical error.

Vaping is highly prevalent, especially amongst adolescents, and its orthopedic effects are poorly understood. We presented three cases as a clinical observation that served as a hypothesis generator. We conducted a PRISMA-compliant systematic review that revealed a scarcity of evidence about vaping and musculoskeletal health. This paper serves as a starting point and a call for high-quality investigation into the orthopedic effects of electronic cigarettes.

## Figures and Tables

**Table 1:** Detailed searching strategy.

Search: **((e-cigarette) OR (vaping)) AND ((orthopedics) OR (surgery))**
(“electronic nicotine delivery systems”[MeSH Terms] OR (“electronic”[All Fields] AND “nicotine”[All Fields] AND “delivery”[All Fields] AND “systems”[All Fields]) OR “electronic nicotine delivery systems”[All Fields] OR “e cigarette”[All Fields] OR (“vaped”[All Fields] OR “vaping”[MeSH Terms] OR “vaping”[All Fields] OR “vapes”[All Fields])) AND (“orthopaedic”[All Fields] OR “orthopedics”[MeSH Terms] OR “orthopedics”[All Fields] OR “orthopedic”[All Fields] OR “orthopaedical”[All Fields] OR “orthopedical”[All Fields] OR “orthopaedics”[All Fields] OR (“surgery”[MeSH Subheading] OR “surgery”[All Fields] OR “surgical procedures, operative”[MeSH Terms] OR (“surgical”[All Fields] AND “procedures”[All Fields] AND “operative”[All Fields]) OR “operative surgical procedures”[All Fields] OR “general surgery”[MeSH Terms] OR (“general”[All Fields] AND “surgery”[All Fields]) OR “general surgery”[All Fields] OR “surgery s”[All Fields] OR “surgerys”[All Fields] OR “surgeries”[All Fields]))

**Table 2:** Summary table of included publications.

**Level 1:** Meta-analyses and randomized controlled trials		
Nil		
**Level 2:** Unrandomized trials, cohort studies, scientific experiments		
Basic science	Pywell et al. [10]	Nonclinical human experiment
	Romagna et al. [11]	Cell line experiment
MSK health	Kennedy et al. [12]	Animal experiment
	Reumann et al. [13]	Animal experiment
Dermal healing	Rau et al. [14]	Animal experiment
	Troiano et al. [15]	Animal experiment
**Level 3:** Expert opinion, briefs, reports		
Non-systematic review, orthopedic	Publications [16,17,18]	
Non-systematic review, surgery, and wounds	Publications [19,20,21,22]	
Briefs and opinion	Publications [23,24,25,26,27,28,29,30]	

## Data Availability

No new data were created or analyzed in this study. Data sharing is not applicable to this article.
